# Regions of XY homology in the pig X chromosome and the boundary of the pseudoautosomal region

**DOI:** 10.1186/1471-2156-14-3

**Published:** 2013-01-15

**Authors:** Benjamin M Skinner, Kim Lachani, Carole A Sargent, Nabeel A Affara

**Affiliations:** 1 Department of Pathology, University of Cambridge, Tennis Court Road, Cambridge, CB2 1QP, UK

**Keywords:** X-chromosome, Genome, Porcine, Evolution, Microarray, Pseudoautosomal

## Abstract

**Background:**

Sex chromosomes are subject to evolutionary pressures distinct from the remainder of the genome, shaping their structure and sequence content. We are interested in the sex chromosomes of domestic pigs (*Sus scrofa*), how their structure and gene content compares and contrasts with other mammalian species, and the role of sex-linked genes in fertility. This requires an understanding of the XY-homologous sequence on these chromosomes.

To this end, we performed microarray-based comparative genomic hybridisation (array-CGH) with male and female Duroc genomic DNA on a pig X-chromosome BAC tiling-path microarray. Putative XY-homologous BACs from regions of interest were subsequently FISH mapped.

**Results:**

We show that the porcine PAR is approximately 6.5-6.9 Mb at the beginning of the short arm of the X, with gene content reflective of the artiodactyl common ancestor. Our array-CGH data also shows an XY-homologous region close to the end of the X long arm, spanning three X BACs. These BACs were FISH mapped, and paint the entire long arm of SSCY. Further clones of interest revealed X-autosomal homology or regions containing repetitive content.

**Conclusions:**

This study has identified regions of XY homology in the pig genome, and defined the boundary of the PAR on the X chromosome. This adds to our understanding of the evolution of the sex chromosomes in different mammalian lineages, and will prove valuable for future comparative genomic work in suids and for the construction and annotation of the genome sequence for the sex chromosomes. Our finding that the SSCYq repetitive content has corresponding sequence on the X chromosome gives further insight into structure of SSCY, and suggests further functionally important sequences remain to be discovered on the X and Y.

## Background

Domestic pigs (*Sus scrofa*) are agriculturally important animals, as well as important model organisms for many human diseases. The genome sequencing effort associated with the domestic pig has recently been published [1], with the current Sscrofa10.2 assembly available on the Ensembl database (http://www.ensembl.org/Sus_scrofa/Info/Index). However, this sequence information is limited to the autosomes and the X chromosomes; little sequence data is available for the Y chromosome.

Pig genomes are comprised of 18 autosome pairs, and the sex chromosomes [2]. The X chromosome shows a small interstitial C-band on the short arm and a large G-band on the long arm, thought to correlate with repetitive content [3,4]. A single known pseudoautosomal region (PAR) lies at the terminus of the X short arm. Several genes have been mapped to the pig PAR - for example, *KAL1* and *STS*[5]. Though not in PAR1 of humans, these may be PAR-related in some other mammalian species (e.g. cow and dog [6]). However, the pseudoautosomal boundary (PAB), and thus the gene complement of the pig PAR, remains imprecisely defined. Evidence from the cow, a relatively closely related species, suggests that the PAR may be 5-9 Mb in size [7].

The Y chromosome is the smallest of the pig chromosomes, and information about its structure and gene content has remained elusive since the identification of the pig sex chromosomes [8]. Cytogenetic investigation has shown the pig Y to be a metacentric chromosome, estimated to be about 50 Mb in length by flow cytometry [9]. Early studies looking at the banding pattern of the Y noted that the long arm (Yq) contains a large C band, indicating that this arm contains a substantial proportion of constitutive heterochromatin [3,10]. Subsequent physical mapping of bacterial artificial chromosome (BAC) clones containing Y chromosome content by fluorescence *in-situ* hybridisation (FISH) has revealed that the long arm can be almost entirely painted by a single BAC clone, while most, if not all, of the single copy sequences are found on the short arm [5]. This level of repetitive sequence further complicates attempts to sequence this chromosome, a problem common to mammalian Y chromosomes (e.g. mouse; [11-15]).

Some clues as to the structure of the pig Y have come from the search for male specific DNA sequences. These yielded short repetitive sequences [16-19], which appear male-specific (or at a very high copy number on the Y relative to the autosomes). The sequence identity between these different repeats ranges from 80-91%. Together these sequences presumably partially explain the repetitive nature of SSCYq. The broad organisation of SSCYq seems complex, but it is apparent that it contains an expanding and diverging group of repeats. It remains unknown however whether there are regions of XY homology on the long arm of the Y chromosome interspersed amongst the repetitive content.

Given the above, the aims of this study were therefore to determine the boundary and size of the porcine PAR, and to identify any other regions of XY homology through the use of microarray-based comparative genomic hybridisation to give insights into sex chromosome evolution in mammals in general and pigs in particular.

## Results and discussion

### Regions of XY homology identified by array-CGH

**Figure 1:**
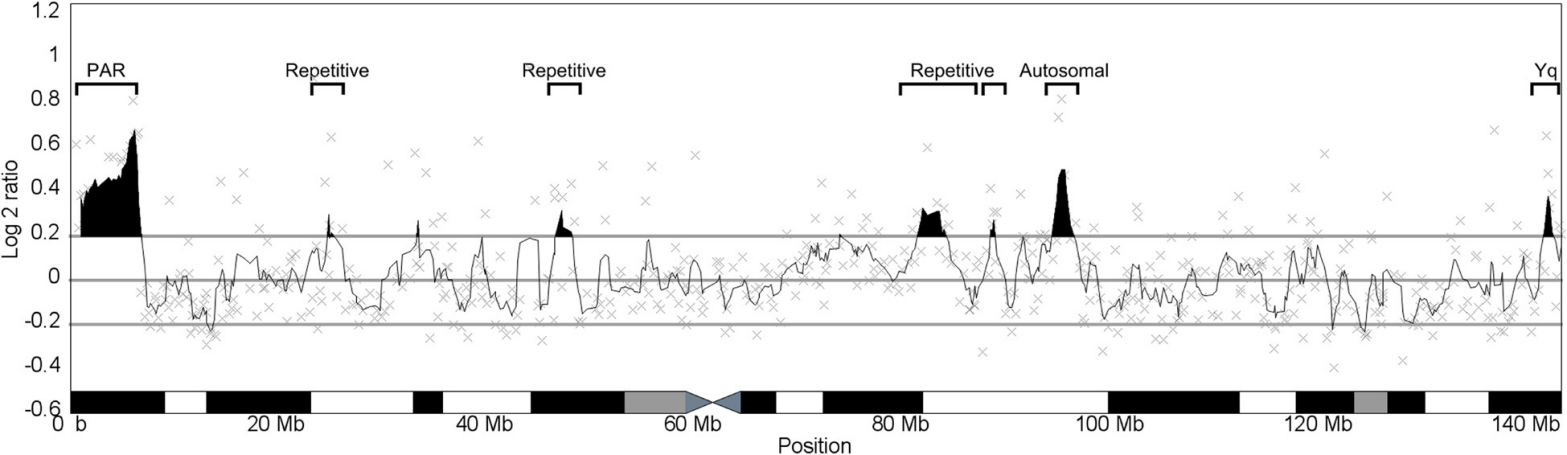
**Array CGH results. **The average log_2_ ratios for each BAC clone along the X chromosome are shown as grey crosses. A 5-clone average has been taken (black line). Regions of interest (log_2_ > 0.2) are highlighted in black, including the PAR and an XY homology block near the end of Xq. The chromosome ideogram is adapted from Ensembl.

Male and female Duroc genomic DNA were competitively hybridised to an X-chromosome tiling-path microarray. The averaged log_2_ ratios from the four technical replicates are shown in Figure 1. This figure also shows a sliding 5-clone window average of log_2_ ratios along the chromosome, and highlights regions of interest with a log_2_ ratio greater than 0.2. Several regions of the chromosome stand out from this view: the p-terminus; the q-terminus; and three regions at approximately 20 Mb, 45 Mb and between 70-80 Mb. The average log_2_ ratios for each clone are given in Additional file 1: Table S1; full array data is deposited at GEO under accession GSE40244 (https://www.ncbi.nlm.nih.gov/geo/query/acc.cgi?acc=GSE40244).

**Figure 2:**
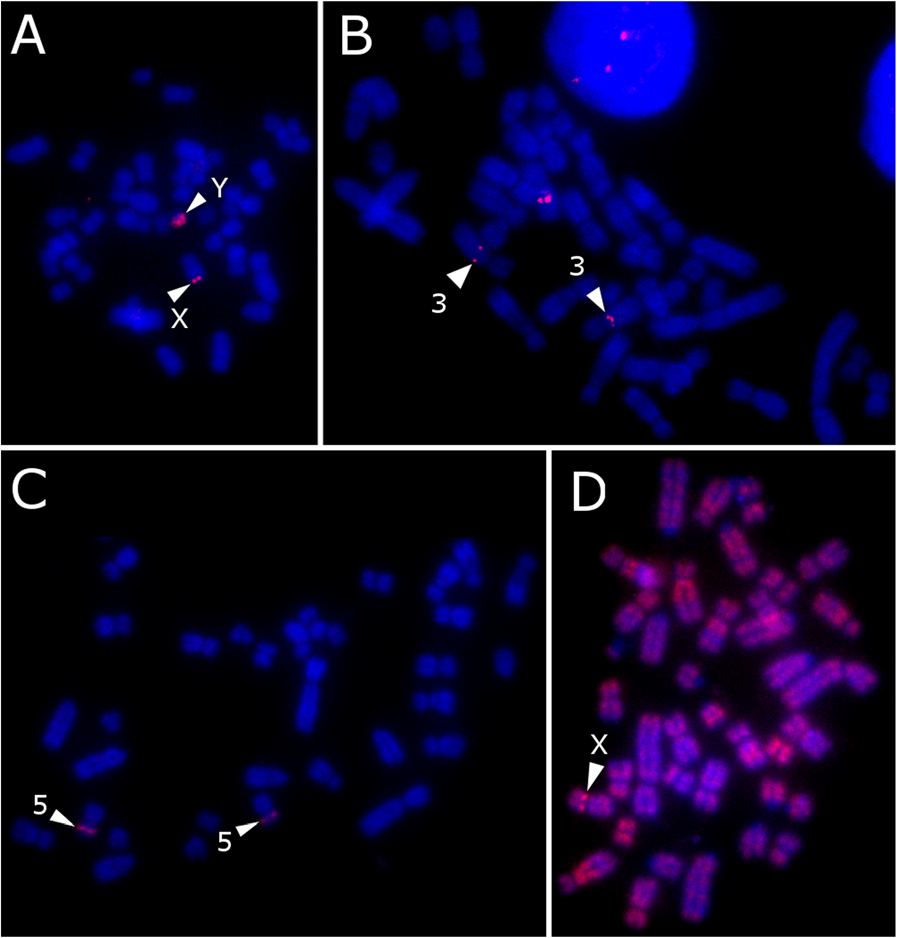
**FISH images. A**) BAC CH242-96N5 on SSCXq also paints all (or most) of SSCYq. **B**) CH242-34G16 hybridises to SSCX and SSC3q **C**) BAC CH242-131F14 hybridises to autosome pair SSC5. **D**) BAC CH242-9O15 from a repetitive region on SSCXq also paints much of the pig genome. Note that background thresholding has been deliberately reduced in this image. Arrows highlight FISH signals in each panel.

BAC clones were selected from regions of interest for FISH mapping on male Duroc chromosomes (from the same male used in the CGH experiments). Some single clones with high log2 ratios were also investigated. Example FISH results from clones in these regions are shown in Figure 2. FISH mapping revealed four types of hybridisation: (1) XY-homologous clones; (2) X-autosomal clones; (3) Pangenomic repetitive clones (4) Autosomal clones. A complete list of successful FISH experiments is given in Additional file 2: Table S2.

### XY-homologous clones

Two regions of XY-homology were found: the PAR, described in greater detail below, and a region towards the q-terminus of Xq. The 3 BACs in this region (CH242-139K22, CH242-115P19 and CH242-96N5) painted the entire long arm of the Y chromosome (Figure 2A) in addition to their discrete X signals. These were flanked proximally by CH242-390A7 and distally by CH242-305A15 (see also Additional file 2: Table S2). Although the microarray results suggest that the XY-homology may continue to the SSCX q-terminus, FISH mapped clones surrounding the XY-homology block appear X-specific.

Pig Yq is known to be highly repetitive [5]. A number of male-specific repetitive sequences have been previously identified [16-19], which must contribute to Yq. However, a comparison by BLAST of these repetitive sequences with the three X BAC clones found in this study failed to show significant matches. That is, whatever sequence underlies the XY homology is not one of the previously identified repeats. Moreover, no BLAST matches were found against any of the clones on the array for which sequence is available. Very little sequence appears to be shared between these 3 BACs in a BAC-BAC BLAST comparison. Those short (~40 bp) sequences that are shared are also found in the surrounding X-specific BACs. Consequently, the precise sequence underlying the XY homology remains undetermined. It does however confirm that the organisation of SSCYq is more complex than tandemly repeated copies of the already-identified male-specific repeats. Further work will be required to determine whether the repetitive long arm of SSCY contains ampliconic genes, as found in the male-specific regions of Y chromosomes of other species (e.g. [13,14,20,21]).

### X-autosomal, autosomal and repetitive clones

One clone, CH242-34G16, showed homology between the X chromosome and the autosome pair SSC3 (Figure 2B). Some pig satellite DNAs are already known to be present on X and autosomes, primarily centromeric satellites – for example, SSCSR1A found at the centromeres of chromosomes 3 and X [22]. Given the position of the clone in a repetitive portion of Xq it seems likely that a similar satellite underlies the homology.

Two other clones that appeared of interest from the microarray experiments physically mapped to autosomes - CH242-100A10 on SSC18 and CH242-131F14 on SSC5p (Figure 2C). These are likely clones miss-assigned during the construction of the library, as they show no evidence for X or Y signal.

Many clones within regions of interest along the X chromosome, for example CH242-9O15, appear to contain a high amount of repetitive content from FISH mapping, despite blocking with Hybloc (Cot-1) DNA (Figure 2D – background thresholding is deliberately reduced). Signals from these probes paint many of the repetitive regions of the pig genome, and this presumably explains their high log_2_ ratios. Supporting this idea, the repetitive region on SSCXp appears to coincide with a known heterochromatic C-band, and the region on SSCXq correlates with a prominent G-band [3,4].

### Boundary and gene content of the porcine PAR

**Figure 3:**
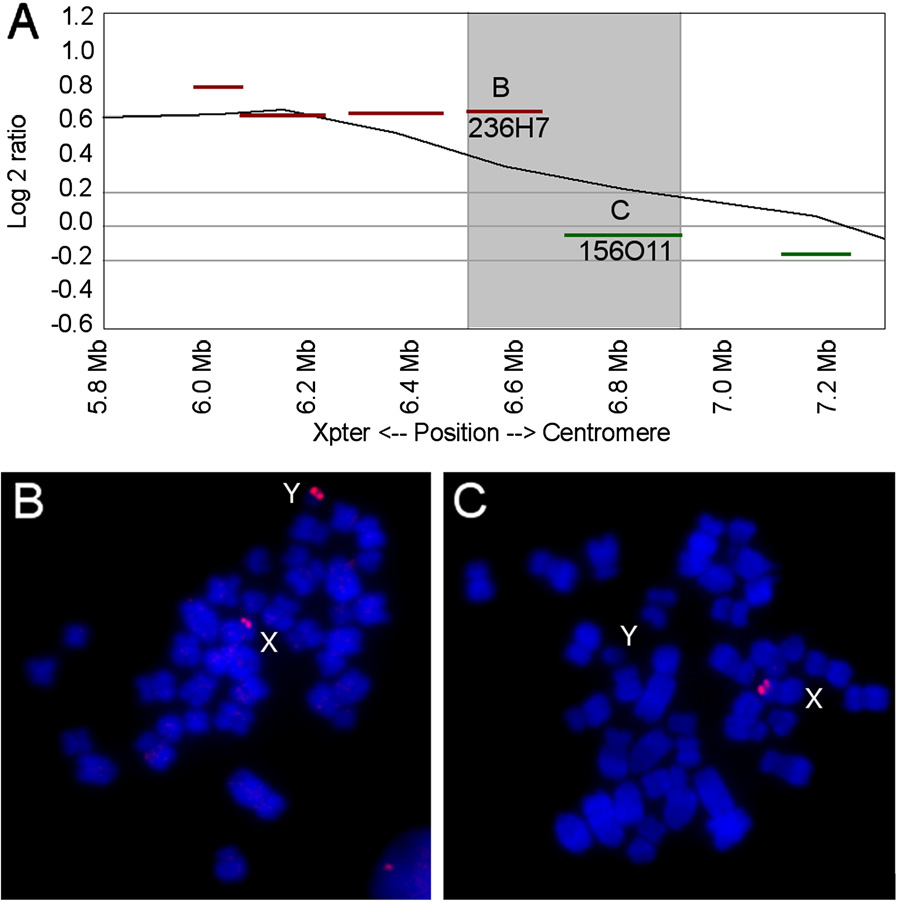
**PAR boundary. **Determination of the PAR boundary. **A**) Log_2_ plot as per Figure 1, focussed on the PAR boundary region. The black line shows the 5-clone average of log_2_ ratios. Red and green lines show the approximate region encompassed by individual BACs in the tiling path, at appropriate log_2_ positions. Red lines show BACs confirmed as XY-homologous by FISH; green lines show X-only BACs. The PAR boundary must lie within the highlighted ~400 kb region of 6.5 Mb to 6.9 Mb. **B**) FISH image of the last PAR BAC (CH242-236H7). **C**) FISH image of the subsequent X-specific BAC (CH242-156O11).

Microarray results suggest that the pseudoautosomal boundary (PAB) lies close to the boundary of clones CH242-236H7 and CH242-156O11. FISH experiments with clones in this region show a clear X and Y signal for BAC 236H7, and an X signal only for BAC 156O11 (Figure 3). Based on the map positions available for these clones (Sscrofa10.2, http://www.ensembl.org/Sus_scrofa/Info/Index), this narrows the PAB on SSCX to a region of ~400 kb between 6.50 Mb and 6.91 Mb. We used the currently available SSCY fosmid sequences to constrain the PAB further by BLAST. A number of fosmids map across 236H7 with 99% sequence identity. Two fosmids map into 156O11 with 99% sequence identity. Of these, the most distal is WTSI_1061-74L5 (accession FO082688, sequencing still in progress at time of writing). This would place the PAB within or around the gene *SHROOM2*. *SHROOM2* mRNA maps partly within 74L5, extending distally by ~40 kb. Consequently, the PAB likely occurs within or shortly proximal to SHROOM2.

The PAR size of ~6.7 Mb is comparable to that of other mammalian PARs that have been sequenced. It is most similar to cattle (5-9 Mb [7]; by Ensemble, about 5.5-6 Mb) and dog (6.6 Mb [23]). It is longer than the PARs of horse (1.8 Mb [24]), human PAR1 (2.7Mb [25]) chimp (2.7 Mb [26-28]) or mouse (700 kb [29]). Although the sizes and gene content of PARs change over time as sex-specific regions expand, and autosomal regions are acquired onto the sex chromosomes (e.g. [24,30]), it appears that gross changes to the PAB may have occurred infrequently in the cetartiodactyl lineages.

A PAB proximal to *SHROOM2-like* in pig is consistent with mapping of PABs in cow and dog; these species PABs are in the interval between *SHROOM2* and *GPR143* in cow [31] and distal to *SHROOM2* in dog [23]. Furthermore, evidence also indicates that *SHROOM2* and *GPR143* are pseudoautosomal in porpoise [31] suggesting that the pig has retained much, but not all, of the ancestral cetartiodactyl PAR.

**Figure 4:**
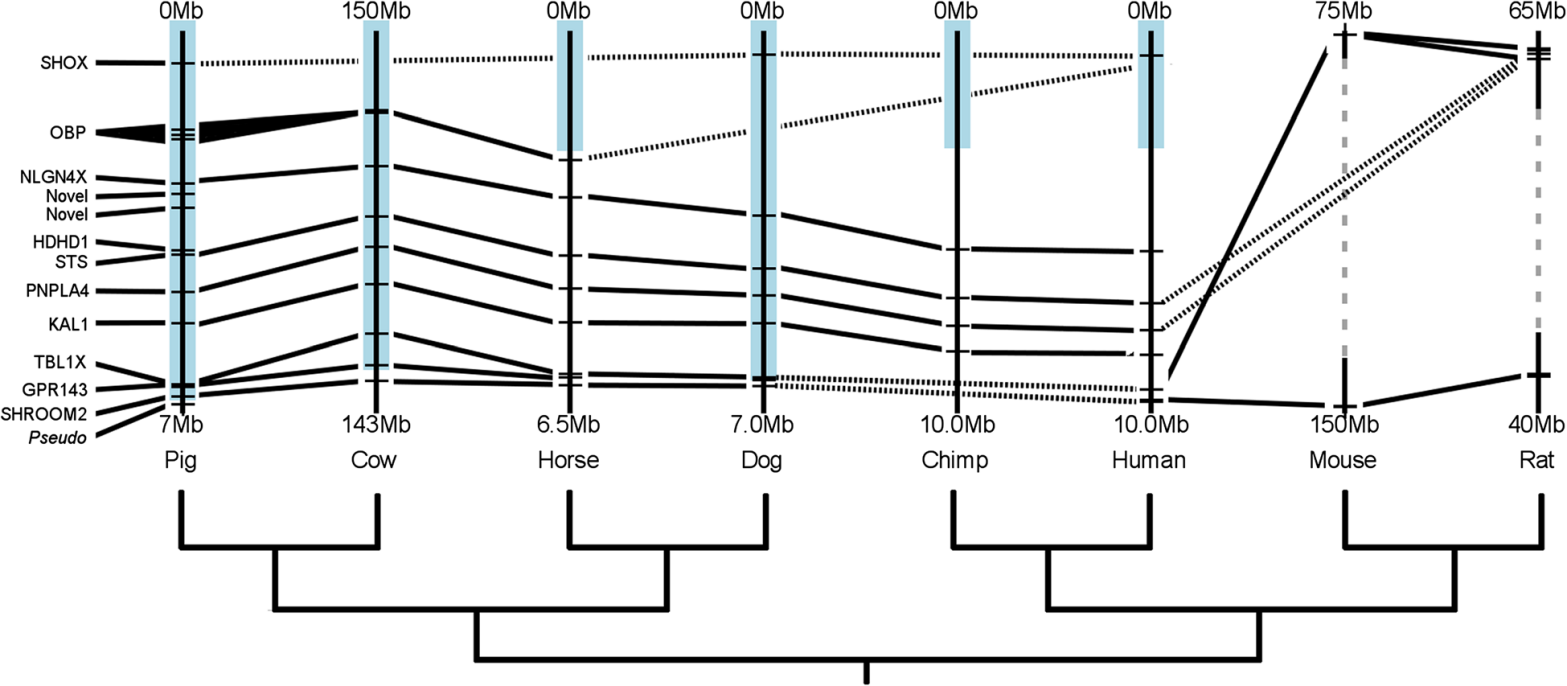
**Comparison of pig PAR gene content across species. **The currently known genes in the pig PAR are shown based on the boundary established in this study. Orthologous genes are shown in other mammalian lineages as available in Ensembl (http://www.ensembl.org). A phylogeny is shown below following [1]. The pig PAR appears to reflect the ancestral artiodactyl PAR based on similarity to cow and the closest outgroup, dog; the horse PAR has contracted [24]. Blue outlines represent the approximate extent of the PAR in each species. Solid lines link orthologous genes from adjacent species; dotted lines link orthologous genes between non-adjacent species.

Genes previously been mapped to the pig PAR include genes known to be XY homologous in many other mammals (e.g. *KAL1*, *STS*[5]). The known genes from Ensembl (http://www.ensembl.org) within the PAR as defined in this study are presented in Figure 4 with orthologous genes from other mammalian species for which X chromosome data is available (full data in Additional file 3: Table S3). Note that the figure shows only the orthologues of genes described in pig - other species-specific genes within these intervals are not represented here. Gene order is broadly conserved in all species but the rodents. Mouse and rat PARs are known to be non-homologous to the human PAR; hence it is unsurprising that there is no overlap between pig and mouse/rat PARs [28]. *PRKX* has previously been reported to be within the PAR [5]. This gene is found within BAC clone CH242-231E5, currently assigned to the end of the X long arm. FISH mapping revealed the BAC is indeed in the PAR, but *PRKX* is not included in Figure 4, as the position within the PAR remains unknown.

Many of the genes that are seen in the pig PAR have clear homologues in other mammalian species. However, variation is seen; there appears to have been an expansion of odorant-binding genes in pig in a cluster - multiple orthologous genes are also seen in mouse and rat, and perhaps cow, but not in the other species. The gene *SHOX*, seen in the PARs of pig, dog and human only here, has been secondarily lost from the sex chromosomes in mice and rats [32], and presumably from cow also. The horse *SHOX* cDNA, together with cDNA sequences for predicted X-genes *PLCXD1* and *PPP2R3B*, is found on the unmapped sequence chromosome Un0036. These genes have been confirmed PAR associated in horse [24]. This suggests that the horse X assembly is lacking a region at the tip of the short arm, corresponding to Un0036.

There are two novel protein-coding genes in pig shown in Figure 4. When the protein sequences for these genes are compared to the NCBI nr database by BLAST (http://blast.ncbi.nlm.nih.gov/Blast.cgi), they both most closely match neuroligin-4-X or X-like variants. Since *NLGN4X* currently covers 465.6 kb of genomic sequence, these may represent errors in the current X assembly. Otherwise, they may reflect transcript variants or gene expansions unique to the suid lineage. *NLGN4X* is not found within PAR1 of humans, nor is it within the PARs of chimp, horse or mouse. It is however within the PARs of cattle and dog. This reflects the differences in PAR boundary locations in these species, and the broadly conservation of gene order on mammalian X chromosomes. Given the importance of neuroligins in synaptogenesis, and their association with human mental disorders such as autism [33], any functional effects of these genes in pigs would be of interest.

Genes on the human PAR1 (accessed from Ensembl) were compared against the pig genome using BLAT (http://www.ensembl.org/Sus_scrofa/blastview). Of 16 genes, 9 had no match, 3 had a closest match on an autosome, 2 had a closest match on the X outside the pig PAR, and 2 were within the pig PAR (Table 1).

**Table 1:** Human PAR genes and their pig orthologues

**Ensembl ID**	**Description**	**Symbol**	**Pig closest match by BLAT**
ENSG00000182378	phosphatidylinositol-specific phospholipase C, X domain containing 1	*PLCXD1*	No match
ENSG00000178605	GTP binding protein 6 (putative)	*GTPBP6*	No match
ENSG00000167393	protein phosphatase 2, regulatory subunit B”, beta	*PPP2R3B*	SSC13
ENSG00000185960	short stature homeobox	*SHOX*	*SHOX*
ENSG00000205755	cytokine receptor-like factor 2	*CRLF2*	No match
ENSG00000198223	colony stimulating factor 2 receptor, alpha, low-affinity (granulocyte-macrophage)	*CSF2RA*	No match
ENSG00000185291	interleukin 3 receptor, alpha (low affinity)	*IL3RA*	No match
ENSG00000169100	solute carrier family 25 (mitochondrial carrier; adenine nucleotide translocator), member 6	*SLC25A6*	*F2Z565_PIG - SSCX, 96.7 Mb*
ENSG00000169093	acetylserotonin O-methyltransferase-like	*ASMTL*	No match
ENSG00000182162	purinergic receptor P2Y, G-protein coupled, 8	*P2RY8*	PAR sequence ~446 kb, no gene
ENSG00000197976	A kinase (PRKA) anchor protein 17A	*AKAP17A*	SSC1, 16
ENSG00000196433	acetylserotonin O-methyltransferase	*ASMT*	No match
ENSG00000169084	dehydrogenase/reductase (SDR family) X-linked	*DHRSX*	SSCX, 89 Mb. SSC5, 2
ENSG00000214717	zinc finger, BED-type containing 1	*ZBED1*	SSC9, 1, 8, no gene
ENSG00000002586	CD99 molecule	*CD99*	No match
ENSG00000124343	Xg blood group	*XG*	No match

Data from Ensembl. Very few of the human PAR genes have as yet identified PAR orthologues in pig.

It is likely that as the pig X sequence annotation improves, some of the ‘missing’ genes will be found, and some of the novel sequences will be found to be errors in the automated annotation, or assigned homologues. Those that remain represent interesting targets for future studies in pig development and evolutionary history. Of equal interest will be genes that are within the PAR in some mammalian species, and not in others (e.g. *NLGN4X).* These may be subject to rapid divergence between the X and Y variants, and hence have acquired important functional differences.

## Conclusion

This study has identified regions of XY homology in the pig genome, and defined the boundary of the PAR on the X chromosome. This adds to our understanding of the evolution of the sex chromosomes in different mammalian lineages, and will prove valuable for future comparative genomic work in suids and for the construction and annotation of the genome sequence for the sex chromosomes. Our finding that the SSCYq repetitive content has corresponding sequence on the X chromosome gives further insight into structure of SSCY, and suggests the potential for other, perhaps functional, material embedded within.

## Methods

### Array-CGH

Microarray construction followed the protocol of Fiegler et al. [34]. Briefly, the BAC clones of the tiling path were grown overnight from glycerol stocks in LB in 96 well deep-well plates. BAC DNA was extracted by mini-prep, purified via 96-well filter-plates and eluted in ddH_2_O. The purified products were amplified by DOP-PCR. Amplified DOP-PCR products were tagged with an aminolinker by PCR, filter-purified and eluted in spotting buffer. Amino-linked products were spotted onto CodeLink microarray slides using an MGII610 (BioRobotics).

### Probe labelling and hybridisation

Genomic DNAs from male and female Duroc pigs were labelled with Cy3 and Cy5 by random priming following [34] with the following modifications: 1 μg Porcine Hybloc DNA (a Cot-1 equivalent - Applied Genetics Laboratories, Florida) was used as a competitor DNA with 0.15 μg genomic DNA. Genomic DNA was extracted using a Qiagen Blood and Tissue Kit. Male DNA was obtained from muscle provided by Genus, from a Duroc boar being routinely culled. Female gDNA was extracted from Duroc sow fibroblast cell line 2–14 provided by Fengtang Yang at the Wellcome Trust Sanger Institute. Hybridisation was performed in a moist chamber for 48 hours. Post-hybridisation washes followed [34].

### Imaging and analysis

Each microarray slide was scanned using a GenePix Personal 4100A Scanner (Axon Instruments) at a 5 μm resolution. Cy3 and Cy5 fluorescence intensities of each DNA spot was quantified in BlueFuse for Microarrays v3.2 (Blue-Gnome Ltd, Cambridge, UK). The Cy3/Cy5 fluorescence ratios were calculated and transformed into log_2_ values. The ratios were normalized using block median normalization for each of the sixteen blocks to account for any block-block variation. The results from the four experiments were exported from the software for further analysis using Inforsense 5.0.1. Each hybridization was treated as a separate experiment. Clones with a confidence score < 0.7 in each experiment were excluded. An average was taken of the log_2_ ratios for each clone across the experiments. Clones were further filtered to leave only those 586 clones with surviving log_2_ ratios in 2 or more experiments. Full results from the array experiments are available on GEO with accession GSE40244; the average log_2_ ratios for each clone are also presented in Additional file 1: Table S1. At time of writing, most clones are sequenced and at least partially assembled. Information may be found on clone status at NCBI (http://www.ncbi.nlm.nih.gov/nuccore).

Approximate start and stop positions were downloaded from the Sscrofa10.2 assembly on Ensemble for each clone to enable ordering of the clones. A 5-clone average was taken along the length of the chromosome to assist visualisation. Clone position was plotted against log_2_ ratio using gnuplot (http://www.gnuplot.org). The SSCX ideogram was adapted from the Pre-Ensemble cytogenetic map (http://pre.ensembl.org/Sus_scrofa_map/Location/Genome Ensemble).

#### Fluorescent in-situ hybridisation (FISH)

Chromosome preparations were made from a fibroblast cell line derived from a skin biopsy of the same male Duroc used for array-CGH experiments. The cell line was established at the Well come Trust Sanger Institute. Chromosome preparation followed standard protocols [35,36]: mitostatic treatment with colcemid at a final concentration of 0.1 μg/ml for 1 h at 37°C, hypotonic treatment with 75 mM KCl for 15 min at 37°C and fixation with 3:1 methanol:acetic acid.

BACs of interest from array-CGH were labelled by nick translation with biotin-16-dUTP (Roche) following standard protocols. Slides with metaphase preparations were aged overnight at 37°C then treated with 4 mg/ml RNase A for one hour at 37°C. The chromosomes were denatured for 1 minute 30 seconds in 70% formamide in 2 × SSC at 70°C. BACs were applied to slides and sealed under coverslips with rubber cement. Hybridization was carried out in a humidified chamber for 24 hours at 37°C. Following post-hybridization washes (40% formamide in 2 × SSC for 20 minutes; 1 minute in 2 × SSC/0.1% Igepal at RT; 15 minutes in 4 × SSC/0.05% Tween 20 at RT; 25 minutes in 4 × SSC/0.05% Tween 20/2% BSA at RT), probes were detected with 1:200 streptavidin-Cy3 (Amersham) in 4 × SSC, 0.05% Igepal, 1.25% BSA for 35 minutes. Slides were washed in 4 × SSC, 0.05% Igepal for 2 × 5 minutes then counterstained using Vectashield with DAPI (Vector Labs). Slides were analyzed on Nikon Microphot-SA epifluorescence microscope equipped with a cooled CCD camera and appropriate filters. Images were captured using SmartCapture 2 (Digital Scientific UK).

## Competing interests

The authors report no competing interests.

## Authors’ contributions

NA and CAS conceived and designed the study and helped draft the manuscript. BMS and KL performed the microarray and FISH experiments. BMS performed data analysis and wrote the manuscript. All authors read and approved the final manuscript.

## Supplementary Material

Additional file 1 **Table S1. **Summary of the microarray results. The clone details, average log_2_ ratios, and positions in the current X assembly are shown. Currently assigned genes are listed by each clone in the PAR. Clones described in the text are highlighted. Click here for file

Additional file 2 **Table S2. **FISH results for selected clones. Clones described in the text are highlighted. Click here for file

Additional file 3 **Table S3. **Multiple mammalian orthologues of pig PAR genes, showing Ensembl gene ids and locations for each orthologue. Species are separated by shading patterns. Some information on X and Y gametologues is included directly from Ensembl, and supplemented by information from [20,21]. Click here for file
